# Synthesis and vinyl benzene copolymerization of novel trisubstituted ethylenes: 15. Halogen and methoxy ring-substituted isopropyl 2-cyano-3-phenyl-2-propenoates

**DOI:** 10.1080/15685551.2020.1782556

**Published:** 2020-06-23

**Authors:** Gregory B. Kharas, Alessandra Cimino, Sebastian Flieger, Paige M. Whelpley, David Ebner, Randi Groy, Christopher R. Savittieri, Nita Shinde, Kenneth L. Thomas, Sara M. Rocus, William S. Schjerven

**Affiliations:** Chemistry and Biochemistry Department, DePaul University, Chicago, IL, USA

**Keywords:** Isopropyl cyanopropenoates, knoevenagel condensation, radical copolymerization, vinyl benzene copolymers

## Abstract

Condensation of isopropyl cyanoacetate and substituted benzoic aldehydes resulted in formation of novel isopropyl esters of 2-cyano-3-phenyl-2-propenoic acid, RPhCH = C(CN)CO_2_CH(CH_3_)_2_ (where R is 2,3,4-trimethoxy, 2,4,5-trimethoxy, 2,4,6-trimethoxy, 3-bromo-4,5-dimethoxy, 5-bromo-2,3-dimethoxy, 5-bromo-2,4-dimethoxy, 6-bromo-3,4-dimethoxy, 2-bromo-3-hydroxy-4-methoxy, 4-bromo-2,6-difluoro, 2-chloro-3,4-dimethoxy, 3-chloro-4,5-dimethoxy, 5-chloro-2,3-dimethoxy, 2,3,6-trichloro, 3-chloro-2,6-difluoro, 2,3,4-trifluoro, 2,4,5-trifluoro, 2,4,6-trifluoro, 3,4,5-trifluoro, 2,3,5,6-tetrafluoro, 2,3,4,5,6-pentafluoro). Copolymerization of the esters with vinyl benzene in solution with radical initiation (ABCN) at 70°C led to formation copolymers. The products were characterized by CHN elemental analysis, IR, ^1^ H- and ^13^ C-NMR, GPC, DSC, and TGA.

## 1. Introduction

Trisubstituted ethylenes, halogen and methoxy ring-substituted cyanophenyl propenoates (CPP), R^1^PhCH = C(CN)CO_2_R^2^ is a large group of functional compounds that found multiple applications in organic and polymer synthesis. Pharmaceutical research is reported for a number of CPP compounds [1–6]. Thus, 2,3,4-trimethoxyphenyl ethyl CPP was involved in highly efficient synthesis of pyronoquinoline [1], whereas 2,4,5–trimethoxy ring–substituted ethyl CPP was used in synthesis and biological screening of some novel pyrozolones and thiazolopyrimidines [2], antitumor activity of novel pyridine, thiophene, thiazole derivatives [3], and potentially antitumorigenic polycyclic chromones and flavones [4]. 2,4,6–Trimethoxyphenyl and 2-bromo-3-hydroxy-4-methoxyphenyl–substituted isopropyl CPP were reported as a cell-active inhibitor of the cancer-promoting phosphatases of regenerating liver [5]. 3-Bromo-4,5-dimethoxyphenyl ethyl CPP was involved in discovery of 4-aryl-2-oxo-2 H-chromenes as a new series of apoptosis inducers [6]. 2,3,4,5,6-Pentafluorophenyl butyl [7] and isobutyl [8] CPP was applied in palladium-catalyzed olefination and arylation of polyfluoroarenes. Electrophilic tri-and tetrasubstituted CPP are useful in demarcating the transition from radical to ionic chemistry [9]. Most CPP compounds do not undergo homopolymerization because of steric difﬁculties but copolymerize readily with monosubstituted alkenes [10]. Trisubstituted alkenes substituted with carbonyl, cyano, and halo groups when copolymerized with monomers like vinyl benzene, *N*-vinylcarbazole, and vinyl acetate [11–13] form alternating copolymers with isolated CPP monomer units. When copolymerized with such commercial monomers, (i.e., vinyl benzene, vinyl, acetate, and vinyl ethers), CPP monomers introduce into polymer chain a variety of functional groups, like cyanoacrylate, substituted phenyl ring, *etc*. These reactive groups could participate in polymer modification reactions to better polymer properties and wider polymer applicability [14].

We continue to explore synthesis and copolymerization of CPP compounds; thus, we have prepared and copolymerized with vinyl benzene ring-trisubstituted methyl [15], ethyl [16], propyl [17,18], and butyl [19,20] esters of 2-cyano-3-phenyl-2-propenoic acid. Recently we have reported synthesis and vinyl benzene copolymerization of a number of novel chloro and methoxy [21] and dimethoxy [22] ring-disubstituted isopropyl esters of 2-cyano-3-phenyl-2-propenoic acid. In this study we have prepared and copolymerized novel ring-substituted isopropyl esters of 2-cyano-3-phenyl-2-propenoic acid, RPhCH = C(CN)CO_2_CH(CH_3_)_2_, ICPP, where R is 2,3,4-trimethoxy, 2,4,5-trimethoxy, 2,4,6-trimethoxy, 3-bromo-4,5-dimethoxy, 5-bromo-2,3-dimethoxy, 5-bromo-2,4-dimethoxy, 6-bromo-3,4-dimethoxy, 2-bromo-3-hydroxy-4-methoxy, 4-bromo-2,6-difluoro, 2-chloro-3,4-dimethoxy, 3-chloro-4,5-dimethoxy, 5-chloro-2,3-dimethoxy, 2,3,6-trichloro, 3-chloro-2,6-difluoro, 2,3,4-trifluoro, 2,4,5-trifluoro, 2,4,6-trifluoro, 3,4,5-trifluoro, 2,3,5,6-tetrafluoro, 2,3,4,5,6-pentafluoro. To the best of our knowledge, there have been no studies of either synthesis of these esters (except synthesis of R = 2,4,6-trimethoxy [5]) nor their copolymerization with vinyl benzene [23].

## 2. Experimental

### 2.1. Materials

2,3,4-trimethoxy, 2,4,5-trimethoxy, 2,4,6-trimethoxy, 3-bromo-4,5-dimethoxy, 5-bromo-2,3-dimethoxy, 5-bromo-2,4-dimethoxy, 6-bromo-3,4-dimethoxy, 2-bromo-3-hydroxy-4-methoxy, 4-bromo-2,6-difluoro, 2-chloro-3,4-dimethoxy, 3-chloro-4,5-dimethoxy, 5-chloro-2,3-dimethoxy, 2,3,6-trichloro, 3-chloro-2,6-difluoro, 2,3,4-trifluoro, 2,4,5-trifluoro, 2,4,6-trifluoro, 3,4,5-trifluoro, 2,3,5,6-tetrafluoro, 2,3,4,5,6-pentafluorobenzoic aldehydes, isopropyl cyanoacetate, piperidine, vinyl benzene, 1,1ʹ-azobiscyclohexanecarbonitrile, (ABCN), and toluene supplied from Sigma-Aldrich Co., were used as received.

### 2.2. Instrumentation

Infrared spectra of the TSE monomers and polymers (NaCl plates) were determined with an ABB FTLA 2000 FT-IR spectrometer. The melting points of the monomers, the glass transition temperatures (*T*_g_), of the copolymers were measured with TA (Thermal Analysis, Inc.) Model Q10 differential scanning calorimeter (DSC). The thermal scans were performed in a 25 to 200ºC range at heating rate of 10ºC/min. The thermal stability of the copolymers was measured by thermogravimetric analyzer (TGA) TA Model Q50 from ambient temperature to 800ºC at 20ºC/min. The molecular weights of the polymers were determined relative to polystyrene standards in THF solutions with sample concentrations 0.8% (w/v) by gel permeation chromatography (GPC) using an Altech 426 HPLC pump at an elution rate of 1.0 mL/min; Phenogel 5μ Linear column at 25ºC and Viscotek 302 detector. ^1^ H- and ^13^ C-NMR spectra were obtained on 10–25% (w/v) monomer or polymer solutions in CDCl_3_ at ambient temperature using Avance 300 MHz spectrometer. Elemental analyses, CHN (wt%) for ICPP compounds, and nitrogen (wt%) for the copolymers were determined accurately to 0.3% for analysis by Midwest Microlab, LLC (IN).

## 3 Results and discussion

### 3.1 Synthesis of ring-substituted isopropyl esters of 2-cyano-3-phenyl-2-propenoic acid

The isopropyl esters of 2-cyano-3-phenyl-2-propenoic acid (ICPA) were synthesized by piperidine catalyzed Knoevenagel condensation [24] of an appropriate benzoic aldehyde with isopropyl cyanoacetate.

RPhCHO + NCCH_2_CO_2_CH(CH_3_)_2_ → RPhCH = C(CN)CO_2_CH(CH_3_)_2_

where R is 2,3,4-trimethoxy, 2,4,5-trimethoxy, 2,4,6-trimethoxy, 3-bromo-4,5-dimethoxy, 5-bromo-2,3-dimethoxy, 5-bromo-2,4-dimethoxy, 6-bromo-3,4-dimethoxy, 2-bromo-3-hydroxy-4-methoxy, 4-bromo-2,6-difluoro, 2-chloro-3,4-dimethoxy, 3-chloro-4,5-dimethoxy, 5-chloro-2,3-dimethoxy, 2,3,6-trichloro, 3-chloro-2,6-difluoro, 2,3,4-trifluoro, 2,4,5-trifluoro, 2,4,6-trifluoro, 3,4,5-trifluoro, 2,3,5,6-tetrafluoro, 2,3,4,5,6-pentafluoro. Thus, equimolar amounts of isopropyl cyanoacetate and an appropriate benzoic aldehyde were mixed in equimolar ratio with a few drops of piperidine. The synthesis proceeded at r.t. for 10 h. The products of the reaction were puriﬁed by recrystallization from isopropanol.

#### 3.1.1. Isopropyl 2-cyano-3-(2,3,4-trimethoxyphenyl)-2-propenoate

Yield 87%; mp 77.3°C, ^1^ H-NMR *δ* 8.6 (s, 1 H, CH =), 8.2–6.7 (m, 2 H, Ph), 5.2 (m, 1 H, OCH), 4.2–3.7 (m, 9 H, OCH_3_), 1.4–1.1 (d, 6 H, CH_3_); ^13^ C-NMR *δ* 163 (C =O), 155 (HC =), 158, 149, 142, 125, 119, 107 (Ph), 116 (CN), 101 (C =), 70 (OCH), 62, 61, 56 (CH_3_O), 22 (CH_3_); IR (cm^−1^): 3049–2732 (m, C-H), 2220 (m, CN), 1722 (s, C =O), 1585 (s, C =C), 1288 (s, C-O-C), 1009, 953 (s, C-H out of plane). Anal. Calcd. for C_16_ H_19_NO_5_: C, 62.94; H, 6.27; N, 4.59; Found: C, 62.52; H, 6.47; N, 3.71.

#### 3.1.2. Isopropyl 2-cyano-3-(2,4,5-trimethoxyphenyl)-2-propenoate

Yield 76%; mp 109.7°C, ^1^ H-NMR *δ* 8.7 (s, 1 H, CH =), 8.1–6.3 (m, 2 H, Ph), 5.2 (m, 1 H, OCH), 4.1–3.8 (m, 9 H, OCH_3_), 1.4 (d, 6 H, CH_3_); ^13^ C-NMR *δ* 163 (C =O), 155 (HC =), 158, 156, 148, 143, 112, 100 (Ph), 117 (CN), 198 (C =), 70 (OCH), 56 (CH_3_O), 22 (CH_3_); IR (cm^−1^): 3023–2782 (m, C-H), 2212 (m, CN), 1695 (s, C =O), 1584 (s, C =C), 1278 (s, C-O-C), 1010, 953, 824 (s, C-H out of plane). Anal. Calcd. for C_16_ H_19_NO_5_: C, 62.94; H, 6.27; N, 4.59; Found: C, 61.98; H, 6.43; N, 3.41.

#### 3.1.3. Isopropyl 2-cyano-3-(2,4,6-trimethoxyphenyl)-2-propenoate

Yield 91%; mp 94.2°C, ^1^ H-NMR *δ* 8.6 (s, 1 H, CH =), 8.2–6.6 (m, 2 H, Ph), 5.1 (m, 1 H, OCH), 3.8 (m, 9 H, OCH_3_), 1.3 (m, 6 H, CH_3_); ^13^ C-NMR *δ* 162 (C =O), 153 (HC =), 157, 149, 143, 126, 119, 108 (Ph), 116 (CN), 140 (C =), 68 (OCH), 55 (CH_3_O), 22 (CH_3_); IR (cm^−1^): 3050–2750 (m, C-H), 2218 (m, CN), 1713 (s, C = O), 1607 (s, C = C), 1288 (s, C-O-C), 1023, 993 (s, C-H out of plane). Anal. Calcd. for C_16_ H_19_NO_5_: C, 62.94; H, 6.27; N, 4.59; Found: C, 62.39; H, 6.41; N, 4.44.

#### 3.1.4. Isopropyl 2-cyano-3-(3-bromo-4,5-dimethoxyphenyl)-2-propenoate

Yield 93%; mp 93.1°C, ^1^ H-NMR *δ* 8.3 (s, 1 H, CH =), 7.7–7,2 (m, 2 H, Ph), 5.1 (m, 1 H, OCH), 3.8 (d, 6 H, OCH_3_), 1.3 (d, 6 H, CH_3_); ^13^ C-NMR *δ* 166 (C = O), 154 (HC =), 152, 129, 128, 117 (Ph), 116 (CN), 100 (C =), 68 (OCH), 61, 56 (CH_3_O), 22 (CH_3_); IR (cm^−1^): 3326–2822 (m, C-H), 2224 (m, CN), 1719 (s, C = O), 1585 (s, C = C), 1247 (s, C-O-C), 953, 838 (s, C-H out of plane). Anal. Calcd. for C_15_H_16_BrNO_4_: C, 50.87; H, 4.55; N, 3.95; Found: C, 51.19; H, 4.64; N, 4.02.

#### 3.1.5. Isopropyl 2-cyano-3-(5-bromo-2,3-dimethoxyphenyl)-2-propenoate

Yield 92%; mp 94.5°C, ^1^ H-NMR *δ* 8.2 (s, 1 H, CH =), 7.7, 7,2 (m, 2 H, Ph), 5.1 (m, 1 H, OCH), 3.8 (d, 6 H, OCH_3_), 1.3 (d, 6 H, CH_3_); ^13^ C-NMR *δ* 166 (C = O), 154 (HC =), 152, 128, 117 (Ph), 116 (CN), 100 (C =), 68 (OCH), 61, 56 (CH_3_O), 22 (CH_3_); IR (cm^−1^): 3344–2822 (m, C-H), 2228 (m, CN), 1729 (s, C = O), 1583 (s, C = C), 1247 (s, C-O-C), 955, 839 (s, C-H out of plane). Anal. Calcd. for C_15_H_16_BrNO_4_: C, 50.87; H, 4.55; N, 3.95; Found: C, 50.44; H, 4.74; N, 4.22.

#### 3.1.6. Isopropyl 2-cyano-3-(5-bromo-2,4-dimethoxyphenyl)-2-propenoate

Yield 87%; mp 166.5°C, ^1^ H-NMR *δ* 8.2 (s, 1 H, CH =), 7.7, 7,2 (m, 2 H, Ph), 5.1 (m, 1 H, OCH), 3.8 (d, 6 H, OCH_3_), 1.3 (d, 6 H, CH_3_); ^13^ C-NMR *δ* 166 (C = O), 154 (HC =), 152, 128, 117 (Ph), 116 (CN), 100 (C =), 68 (OCH), 61, 56 (CH_3_O), 22 (CH_3_); IR (cm^−1^): 3398–2822 (m, C-H), 2214 (m, CN), 1712 (s, C = O), 1564 (s, C = C), 1249 (s, C-O-C), 955, 833 (s, C-H out of plane). Anal. Calcd. for C_15_H_16_BrNO_4_: C, 50.87; H, 4.55; N, 3.95; Found: C, 51.19; H, 4.64; N, 4.16.

#### 3.1.7. Isopropyl 2-cyano-3-(6-bromo-3,4-dimethoxyphenyl)-2-propenoate

Yield 80%; mp 134.8°C, ^1^ H-NMR *δ* 8.1 (s, 1 H, CH =), 7.1, 7,0 (m, 2 H, Ph), 5.1 (m, 1 H, OCH), 3.8 (d, 6 H, OCH_3_), 1.3 (d, 6 H, CH_3_); ^13^ C-NMR *δ* 162 (C = O), 152 (HC =), 153, 148, 128, 117, 114 (Ph), 116 (CN), 128 (C =), 68 (OCH), 56 (CH_3_O), 22 (CH_3_); IR (cm^−1^): 3402–2817 (m, C-H), 2222 (m, CN), 1713 (s, C = O), 1585 (s, C = C), 1232 (s, C-O-C), 957, 821 (s, C-H out of plane). Anal. Calcd. for C_15_H_16_BrNO_4_: C, 50.87; H, 4.55; N, 3.95; Found: C, 51.01; H, 4.64; N, 3.98.

#### 3.1.8. Isopropyl 2-cyano-3-(2-bromo-3-hydroxy-4-methoxyphenyl)-2-propenoate

Yield 79%; mp 126.7°C, ^1^ H-NMR *δ* 10.3 (s, 1 H, OH), 8.2 (s, 1 H, CH =), 7.5, 6,9 (m, 2 H, Ph), 5.1 (m, 1 H, OCH), 3.8 (s, 3 H, OCH_3_), 1.3 (d, 6 H, CH_3_); ^13^ C-NMR *δ* 166 (C = O), 152 (HC =), 153, 128, 127, 114 (Ph), 116 (CN), 129 (C =), 68 (OCH), 56 (CH_3_O), 22 (CH_3_); IR (cm^−1^): 3434–2821 (m, C-H), 2221 (m, CN), 1719 (s, C = O), 1586 (s, C = C), 1254 (s, C-O-C), 958, 842 (s, C-H out of plane). Anal. Calcd. for C_14_H_14_BrNO_4_: C, 49.43; H, 4.15; N, 4.12; Found: C, 48.91; H, 4.31; N, 4.20.

#### 3.1.9. Isopropyl 2-cyano-3-(4-bromo-2,6-diflurophenyl)-2-propenoate

Yield 95%; mp 67.8°C, ^1^ H-NMR *δ* 8.1 (s, 1 H, CH =), 7.2, 7,1 (m, 2 H, Ph), 5.1 (m, 1 H, OCH), 1.3 (d, 6 H, CH_3_); ^13^ C-NMR *δ* 166 (C = O), 152 (HC =), 151, 115 (Ph), 116 (CN), 103 (C =), 68 (OCH), 22 (CH_3_); IR (cm^−1^): 3436–2823 (m, C-H), 2218 (m, CN), 1718 (s, C = O), 1589 (s, C = C), 1231 (s, C-O-C), 956, 821 (s, C-H out of plane). Anal. Calcd. for C_13_H_10_BrF_2_NO_2_: C, 47.30; H, 3.05; N, 4.24; Found: C, 47.88; H, 3.33; N, 4.65.

#### 3.1.10. Isopropyl 2-cyano-3-(2-chloro-3,4-dimethoxyphenyl)-2-propenoate

Yield 79%; mp 126.7°C, ^1^ H-NMR *δ* 8.1 (s, 1 H, CH =), 7.7, 7,5 (m, 2 H, Ph), 5.2 (m, 1 H, OCH), 4.0 (d, 6 H, OCH_3_), 1.4 (d, 6 H, CH_3_); ^13^ C-NMR *δ* 162 (C = O), 154 (HC =), 149, 129, 128, 127, 111 (Ph), 116 (CN), 103 (C =), 71 (OCH), 61, 56 (CH_3_O), 22 (CH_3_); IR (cm^−1^): 3424–2819 (m, C-H), 2218 (m, CN), 1709 (s, C = O), 1580 (s, C = C), 1246 (s, C-O-C), 951, 835 (s, C-H out of plane). Anal. Calcd. for C_15_H_16_ClNO_4_: C, 58.16; H, 5.21; N, 4.52; Found: C, 57.91; H, 5.31; N, 4.40.

#### 3.1.11. Isopropyl 2-cyano-3-(3-chloro-4,5-dimethoxyphenyl)-2-propenoate

Yield 82%; mp 94.5°C, ^1^ H-NMR *δ* 8.6 (s, 1 H, CH =), 8.2–6.7 (m, 2 H, Ph), 5.2 (m, 1 H, OCH), 4.0, 3.9 (s, 6 H, OCH_3_), 1.4 (d, 6 H, CH_3_); ^13^ C-NMR *δ* 162 (C = O), 157 (HC =), 145, 132, 126, 123, 110 (Ph), 116 (CN), 103 (C =), 71 (OCH), 61, 56 (CH_3_O), 22 (CH_3_); IR (cm^−1^): 3150–2802 (m, C-H), 2220 (m, CN), 1717 (s, C = O), 1607 (s, C = C), 1263 (s, C-O-C), 932, 843 (s, C-H out of plane). Anal. Calcd. for C_15_H_16_ClNO_4_: C, 58.16; H, 5.21; N, 4.52; Found: C, 58.00; H, 5.10; N, 4.56.

#### 3.1.12. Isopropyl 2-cyano-3-(5-chloro-2,3-dimethoxyphenyl)-2-propenoate

Yield 77%; mp 103.7°C, ^1^ H-NMR *δ* 8.2 (s, 1 H, CH =), 7.8–7,5 (m, 2 H, Ph), 5.1 (m, 1 H, OCH), 3.8 (d, 6 H, OCH_3_), 1.3 (d, 6 H, CH_3_); ^13^ C-NMR *δ* 166 (C = O), 152 (HC =), 152, 130, 121 (Ph), 116 (CN), 103 (C =), 68 (OCH), 60, 56 (CH_3_O), 22 (CH_3_); IR (cm^−1^): 3443–2809 (m, C-H), 2223 (m, CN), 1718 (s, C = O), 1587 (s, C = C), 1248 (s, C-O-C), 962, 834 (s, C-H out of plane). Anal. Calcd. for C_15_H_16_ClNO_4_: C, 58.16; H, 5.21; N, 4.52; Found: C, 57.21; H, 5.34; N, 4.48.

#### 3.1.13. Isopropyl 2-cyano-3-(2,3,6-trichlorophenyl)-2-propenoate

Yield 98%; mp 85.4°C, ^1^ H-NMR *δ* 8.4 (s, 1 H, CH =), 8.2, 7,2 (s, 2 H, Ph), 5.2 (m, 1 H, OCH), 1.3 (d, 6 H, CH_3_); ^13^ C-NMR *δ* 160 (C = O), 150 (HC =), 133, 132, 120, 128 (Ph), 116 (CN), 126 (C =), 71 (OCH), 21 (CH_3_); IR (cm^−1^): 3423–2829 (m, C-H), 2227 (m, CN), 1722 (s, C = O), 1593 (s, C = C), 1256 (s, C-O-C), 974, 844 (s, C-H out of plane). Anal. Calcd. for C_13_H_10_Cl_3_NO_2_: C, 49.01; H, 3.16; N, 4.40; Found: C, 49.34; H, 3.33; N, 4.59.

#### 3.1.14. Isopropyl 2-cyano-3-(3-chloro-2,6-difluorophenyl)-2-propenoate

Yield 97%; mp 59.4°C, ^1^ H-NMR *δ* 8.1 (s, 1 H, CH =), 7.3, 7,0 (m, 2 H, Ph), 5.1 (m, 1 H, OCH), 1.3 (d, 6 H, CH_3_); ^13^ C-NMR *δ* 166 (C = O), 152 (HC =), 149, 133, 113, 112 (Ph), 116 (CN), 101 (C =), 68 (OCH), 22 (CH_3_); IR (cm^−1^): 3419–2823 (m, C-H), 2221 (m, CN), 1716 (s, C = O), 1576 (s, C = C), 1249 (s, C-O-C), 936, 843 (s, C-H out of plane). Anal. Calcd. for C_13_H_10_ClF_2_NO_2_: C, 54.66; H, 3.53; N, 4.90; Found: C, 54.13; H, 3.83; N, 5.05.

#### 3.1.15. Isopropyl 2-cyano-3-(2,3,4-trifluorophenyl)-2-propenoate

Yield 92%; mp 55.2°C, ^1^ H-NMR *δ* 8.3 (s, 1 H, CH =), 8.2, 7,1 (s, 2 H, Ph), 5.2 (m, 1 H, OCH), 1.3 (d, 6 H, CH_3_); ^13^ C-NMR *δ* 160 (C = O), 152 (HC =), 144, 142, 132, 118, 112 (Ph), 116 (CN), 123 (C =), 70 (OCH), 21 (CH_3_); IR (cm^−1^): 3427–2807 (m, C-H), 2231 (m, CN), 1718 (s, C = O), 1598 (s, C = C), 1259 (s, C-O-C), 965, 843 (s, C-H out of plane). Anal. Calcd. for C_13_H_10_F_3_NO_2_: C, 58.00; H, 3.74; N, 5.20; Found: C, 59.19; H, 4.02; N, 5.46.

#### 3.1.16. Isopropyl 2-cyano-3-(2,4,5-trifluorophenyl)-2-propenoate

Yield 96%; mp 60.3°C, ^1^ H-NMR *δ* 8.3 (s, 1 H, CH =), 8.2, 7,0 (s, 2 H, Ph), 5.2 (m, 1 H, OCH), 1.3 (d, 6 H, CH_3_); ^13^ C-NMR *δ* 160 (C = O), 152 (HC =), 158, 156, 155, 146, 144, 143 (Ph), 116 (CN), 108 (C =), 70 (OCH), 21 (CH_3_); IR (cm^−1^): 3234–2824 (m, C-H), 2226 (m, CN), 1724 (s, C = O), 1578 (s, C = C), 1245 (s, C-O-C), 954, 849 (s, C-H out of plane). Anal. Calcd. for C_13_H_10_F_3_NO_2_: C, 58.00; H, 3.74; N, 5.20; Found: C, 58.75; H, 3.77; N, 5.27.

#### 3.3.17. Isopropyl 2-cyano-3-(2,4,6-trifluorophenyl)-2-propenoate

Yield 78%; mp 64.0°C, ^1^ H-NMR *δ* 8.1 (s, 1 H, CH =), 6.9 (m, 2 H, Ph), 5.2 (m, 1 H, OCH), 1.3 (d, 6 H, CH_3_); ^13^ C-NMR *δ* 163 (C = O), 159 (HC =), 160, 141, 112 (Ph), 116 (CN), 103 (C =), 70 (OCH), 21 (CH_3_); IR (cm^−1^): 3412–2813 (m, C-H), 2236 (m, CN), 1721 (s, C = O), 1576 (s, C = C), 1267 (s, C-O-C), 972, 849 (s, C-H out of plane). Anal. Calcd. for C_13_H_10_F_3_NO_2_: C, 58.00; H, 3.74; N, 5.20; Found: C, 57.88; H, 3.89; N, 5.29.

#### 3.1.18. Isopropyl 2-cyano-3-(3,4,5-trifluorophenyl)-2-propenoate

Yield 82%; mp 96.7°C, ^1^ H-NMR *δ* 8.1 (s, 1 H, CH =), 8.7 (m, 2 H, Ph), 5.2 (m, 1 H, OCH), 1.3 (d, 6H, CH_3_); ^13^ C-NMR *δ* 161 (C = O), 153 (HC =), 151, 150, 144, 141, 128, 118, (Ph), 115 (CN), 106 (C =), 71 (OCH), 22 (CH_3_); IR (cm^−1^): 3446–2907 (m, C-H), 2234 (m, CN), 1721 (s, C = O), 1576 (s, C = C), 1243 (s, C-O-C), 971, 841 (s, C-H out of plane). Anal. Calcd. for C_13_H_10_F_3_NO_2_: C, 58.00; H, 3.74; N, 5.20; Found: C, 58.42; H, 3.84; N, 5.19.

#### 3.1.19. Isopropyl 2-cyano-3-(2,3,5,6-tetrafluorophenyl)-2-propenoate

Yield 93%; mp 63.2°C, ^1^ H-NMR *δ* 8.1 (s, 1 H, CH =), 6.9 (m, 2 H, Ph), 5.2 (m, 1 H, OCH), 1.3 (d, 6H, CH_3_); ^13^ C-NMR *δ* 161 (C = O), 153 (HC =), 145, 141, 128, 115 (Ph), 115 (CN), 106 (C =), 71 (OCH), 22 (CH_3_); IR (cm^−1^): 3422–2834 (m, C-H), 2238 (m, CN), 1722 (s, C = O), 1556 (s, C = C), 1273 (s, C-O-C), 976, 841 (s, C-H out of plane). Anal. Calcd. for C_13_H_9_F_4_NO_2_: C, 58.00; H, 3.74; N, 5.20; Found: C, 54.55; H, 3.45; N, 4.96.

#### 3.1.20. Isopropyl 2-cyano-3-(2,3,4,5,6-pentafluorophenyl)-2-propenoate

Yield 78%; mp 63.2°C, ^1^ H-NMR *δ* 8.0 (s, 1 H, CH =), 5.2 (m, 1 H, OCH), 1.2 (d, 6H, CH_3_); ^13^ C-NMR *δ* 163 (C = O), 152 (HC =), 140, 113 (Ph), 116 (CN), 104 (C =), 71 (OCH), 21 (CH_3_); IR (cm^−1^): 3392–2808 (m, C-H), 2232 (m, CN), 1712 (s, C = O), 1566 (s, C = C), 1282 (s, C-O-C), 967, 832 (s, C-H out of plane). Anal. Calcd. for C_13_H_8_F_5_NO_2_: C, 51.16; H, 2.64; N, 4.59; Found: C, 50.71; H, 3.09; N, 4.97.

### 3.2. Homopolymerization

The IPCA compounds did not homopolymerize on ABCN initiation at 70ºC for 48 h with no polymer precipitated in methanol. Vinyl benzene (VB) polymerization (30 min) resulted in 18.3% yield of polyethenybenzene.

### 3.3. Copolymerization

Copolymers of the vinyl benzene (VB) and the ICPA monomers were prepared at VB/ICPA = 3 (mol) the monomer feed with 0.12 mol/L of ABCN at total monomer concentration 2.44 mol/L in 10 mL of toluene at 70ºC. Polymerization time was 8 h. To stop reaction the mixture was cooled and precipitated in methanol. Nitrogen elemental analysis was used to determine composition of the copolymers. The yield of copolymers was kept low to decrease copolymer compositional drift.

**Scheme 1.**
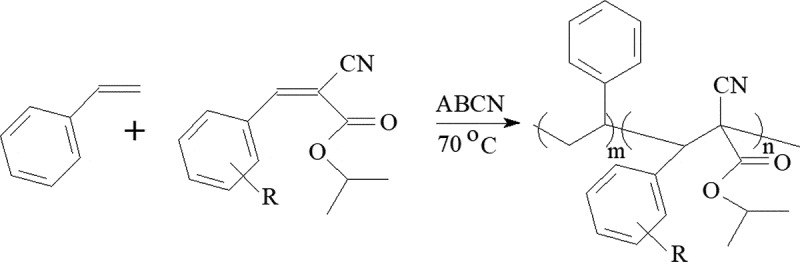
VB-ICPA copolymerization, R = 2,3,4-trimethoxy, 2,4,5-trimethoxy, 2,4,6-trimethoxy, 3-bromo-4,5-dimethoxy, 5-bromo-2,3-dimethoxy, 5-bromo-2,4-dimethoxy, 6-bromo-3,4-dimethoxy, 2-bromo-3-hydroxy-4-methoxy, 4-bromo-2,6-difluoro, 2-chloro-3,4-dimethoxy, 3-chloro-4,5-dimethoxy, 5-chloro-2,3-dimethoxy, 2,3,6-trichloro, 3-chloro-2,6-difluoro, 2,3,4-trifluoro, 2,4,5-trifluoro, 2,4,6-trifluoro, 3,4,5-trifluoro, 2,3,5,6-tetrafluoro, 2,3,4,5,6-pentafluoro

Copolymerization (Sch. 1) of VB and the ring-substituted ICPA resulted in the formation of copolymers (Table 1) with weight-average molecular masses 52 to 61 kD.

**Table 1.** Copolymerization of VB with ICPA

R	N (wt%)	VB in copol (mol%)	ICPA in copol. (mol%)	1/*r*_1_	*M_W_* (kD)
2,3,4-Trimethoxy	2.03	78.7	21.3	1.11	56
2,4,5-Trimethoxy	1.39	87.1	12.9	0.52	56
2,4,6-Trimethoxy	1.67	83.7	16.3	0.73	52
3-Bromo-4,5-dimethoxy	2.45	67.6	32.4	2.75	53
5-Bromo-2,3-dimethoxy	2.46	67.4	32.6	2.81	56
5-Bromo-2,4-dimethoxy	2.44	67.9	32.1	2.69	54
6-Bromo-3,4-dimethoxy	2.14	65.2	34.8	3.43	57
2-bromo-3-hydroxy-4-methoxy	2.45	75.1	24.9	1.48	54
4-Bromo-2,6-difluoro	2.6	66.7	33.3	2.99	58
3-Chloro-2,6-difluoro	2.7	69.1	30.9	2.43	57
2-Chloro-3,4-dimethoxy	1.07	90.6	9.4	0.35	54
3-Chloro-4,5-dimethoxy	2.39	72.6	27.4	1.82	59
5-Chloro-2,3-dimethoxy	2.79	64.8	35.2	3.55	56
2,3,6-Trichloro	2.06	77.6	22.4	1.21	55
2,3,4-Trifluoro	2.21	77.8	22.2	1.20	61
2,4,5-Trifluoro	2.03	81.2	19.8	0.99	59
2,4,6-Trifluoro	1.85	82.4	17.6	0.81	58
3,4,5-Trifluoro	2.19	78.1	21.9	1.17	60
2,3,5,6-tetrafluoro	2.06	79.1	20.9	1.08	58
2,3,4,5,6-Pentafluoro	2.21	76.0	24.0	1.39	59

^a^Polymerization time was 8 h.

Copolymer composition was calculated based of nitrogen analysis in the following way: ICPA (mole) = N (wt %)/14; ICPA (wt%) = ICPA (mole) x Mol. Weight of ICPA;

VB (wt%) = 100 – ICPA (wt%); VB (mol) = VB (wt%)/104; VB (mol %) = VB (mol)/[(ICPA (mol) + VB (mol)] · 100%; ICPA (mol %) = 100 – VB mol %.

According to elemental analysis, between 9.4 and 35.2 mol% of ICPA monomer is present in the copolymers prepared at VB/ICPA = 3 (mol), which is indicative of relatively high reactivity of the monomers towards ST. The copolymers were all soluble in ethyl acetate, THF, DMF and CHCl_3_ and insoluble in methanol, ethyl ether, and petroleum ether.

### 3.4 Monomer relative reactivity

Relative reactivities of VB and the ICPA monomers in the copolymerization can be estimated by application of the copolymerization equation for the terminal copolymerization model [10].

(1) $$m_{1}/m_{2}=\;\left[M_{1}\right]\;(r_{1}\left[M_{1}\right]+\;\left[M_{2}\right])/\left[M_{2}\right]\;(\left[M_{1}\right]+r_{2}\left[M_{2}\right])$$

where *m*_1_ and *m*_2_ are mole fractions of VB and ICPA monomer units in the copolymer, [M_1_] and [M_2_] are concentrations of VB and an ICPA in the monomer feed, and *r*_1_ and *r*_2_ are monomer reactivity ratios, *r*_1_ = *k*_VB-VB_/*k*_VB-ICPA_ and *r*_2_ = *k*_ICPA-ICPA_/*k*_ICPA-VB_. In the absence of self-propagation of ICPA monomers, none of them formed homopolymers, (*k*_ICPA-ICPA_ = 0, *r*_2_ = 0) [10], Eq. (1) yields

(2) $$m_{1}/m_{2}=r_{1}\left(\left[M_{1}\right]/\left[M_{2}\right]\right)+\;1$$

Equation 2 assumes a minimal copolymer compositional drift during a copolymerization reaction, i.e., a low conversion. The fact that ICPA monomers do not self-propagate allows the use of Eq. (2) for a single-point estimation of the relative reactivity of ICPA monomers with respect to VB; it is represented by the 1/*r*_1_ = *k*_VB-ICPA_/*k*_VB-VB_ ratio (the rate constant ratio of attaching an ICPA molecule vs. a VB molecule to a VB-ending growing polymer chain). Taking into account that the [M_1_]/[M_2_] ratio in all the experiments was equal to 3.0, relative reactivities (1/*r*_1_) for the ICPA monomers decrease in the following row R = 5-chloro-2,3-dimethoxy (3.55) > 6-bromo-3,4-dimethoxy (3.43) > 4-bromo-2,6-difluoro (2.99) > 5-bromo-2,3-dimethoxy (2.81) > 3-bromo-4,5-dimethoxy (2.75) > 5-bromo-2,4-dimethoxy (2.69) > 3-chloro-2,6-difluoro (2.43) > 4-chloro-2,6-difluoro (2.10) > 3-chloro-4,5-dimethoxy (1.82) > 2-bromo-3-hydroxy-4-methoxy (1.48) > 2,3,4,5,6-pentafluoro (1.39) > 2,3,6-trichloro (1.21) > 2,3,4-trifluoro (1.20) > 3,4,5-trifluoro (1.17) > 2,3,4-trimethoxy (1.11) > 2,3,5,6-tetrafluoro (1.08) > 2,4,5-trifluoro (0.99) > 2,4,6-trifluoro (0.81) > 2,4,6-trimethoxy (0.73) > 2,4,5-trimethoxy (0.52) > 2-chloro-3,4-dimethoxy (0.35).

These relative reactivity values can be used to predict specific copolymer composition as function of the comonomer feed. Additional research will be needed to correlate effect of phenyl ring substitution with reactivity of ICPA monomers in radical copolymerization.

### 3.5. Thermal behavior

Thermal transitions of the VB-ICPA copolymers were analyzed by differential scanning calorimetry (DSC). The second heating results were obtained in all cases so that the samples become more dry and without ‘thermal memory’. DSC analysis confirmed amorphous morphology of the EB-ICPA copolymers showing glass transition temperatures *T*_g_ and absence of crystalline endotherm on repeated heating and cooling cycles (Table 2). A single *T*_g_ value was observed for all the copolymers with values close to or higher than polystyrene (104ºC) [25]. Introduction of trimethoxy and bromo-dimethoxy phenyl substitution does not change significantly *T*_g_ which is related to segmental mobility [26], whereas chloro-dimethoxy, trifluoro, tetrafluoro, and pentafluro phenyl substitution in VB-ICPA copolymer lead to decrease of segmental mobility in the polymer chain. More precise correlation of the segmental mobility to the size and position of the ICPA ring substitution is difficult apparently due to non-uniform composition, monomer unit distribution, and/or molecular weight and MWD of the copolymers.

**Table 2.** Thermal Behavior of VB – ICPA copolymers

R	*T_g_* (ºC)	Onset of decomp. (ºC)	10 wt% loss (ºC)	50 wt% loss (ºC)	Residue wt%
2,3,4-Trimethoxy	103	225	302	340	2
2,4,5-Trimethoxy	119	258	306	361	2
2,4,6-Trimethoxy	94	222	296	350	2
3-Bromo-4,5-dimethoxy	103	265	302	356	5
5-Bromo-2,3-dimethoxy	107	278	321	374	6
5-Bromo-2,4-dimethoxy	112	265	319	387	6
6-Bromo-3,4-dimethoxy	107	268	316	379	5
2-bromo-3-hydroxy-4-methoxy	108	271	317	345	5
4-Bromo-2,6-difluoro	111	272	314	387	4
3-Chloro-2,6-difluoro	107	267	312	367	5
2-Chloro-3,4-dimethoxy	115	201	310	346	3
3-Chloro-4,5-dimethoxy	157	232	295	345	5
5-Chloro-2,3-dimethoxy	103	262	302	340	2
2,3,6-Trichloro	78	252	270	342	1
2,3,4-Trifluoro	125	263	298	364	1
2,4,5-Trifluoro	141	237	284	335	2
2,4,6-Trifluoro	125	251	296	348	1
3,4,5-Trifluoro	147	222	285	345	2
2,3,5,6-tetrafluoro	151	234	289	355	3
2,3,4,5,6-Pentafluoro	143	174	283	356	7

Thermogravimetric analysis (TGA) provided information on thermal stability of the copolymers (Table 2). Thermal stability of the P(VB-*co*-ICPA) copolymers is lower than that of poly(vinylbenzene, PVB) [27], the onset of decomposition at 219ºC (PVB 350ºC), 10% weight loss at 301ºC (PVB 425ºC), 50% weight loss at 343ºC (PVB 428ºC). Lower thermal stability of the VB-ICPA copolymers apparently associated with presence of ICPA quaternary carbon in the chain similarly to poly-alpha-methylstyrene [28]. TGA showed that the copolymers decomposed in nitrogen in two steps, first in the 200–500ºC range with residue (1–7% wt.), and second in the 500–800ºC range.

## 4. Conclusions

Novel isopropyl esters of ring-substituted 2-cyano-3-phenyl-2-propenoic acid were prepared and copolymerized with vinyl benzene. The compositions of novel copolymers were calculated from nitrogen analysis and the structures were analyzed by IR, H^1^ and ^13^ C-NMR. The thermal gravimetric analysis indicated that the copolymers decompose in two steps, first in the 200–500°C range with a residue, which then decomposed in the 500–800ºC range.
